# Iron‐sulfur cluster ISD11 deficiency (*LYRM4* gene) presenting as cardiorespiratory arrest and 3‐methylglutaconic aciduria

**DOI:** 10.1002/jmd2.12058

**Published:** 2019-07-24

**Authors:** Margarida Paiva Coelho, Joana Correia, Aureliano Dias, Célia Nogueira, Anabela Bandeira, Esmeralda Martins, Laura Vilarinho

**Affiliations:** ^1^ Reference Center for Metabolic Disorders Centro Hospitalar Universitário do Porto Porto Portugal; ^2^ Newborn Screening, Metabolism and Genetics Unit, Human Genetics Department National Institute of Health Doutor Ricardo Jorge Lisboa Portugal

**Keywords:** 3‐methylglutaconic aciduria, Fe‐S clusters, ISD11, *LYRM4*, mitochondrial disorder

## Abstract

In the era of genomics, the number of genes linked to mitochondrial disease has been quickly growing, producing massive knowledge on mitochondrial biochemistry. *LYRM4* gene codifies for ISD11, a small protein (11 kDa) acting as an iron‐sulfur cluster, that has been recently confirmed as a disease‐causing gene for mitochondrial disorders. We present a 4‐year‐old girl patient, born from non‐consanguineous healthy parents, with two episodes of cardiorespiratory arrest after respiratory viral illness with progressive decreased activity and lethargy, at the age of 2 and 3 years. She was asymptomatic between crisis with regular growth and normal development. During acute events of illness, she had hyperlactacidemia (maximum lactate 5.2 mmol/L) and urinary excretion of ketone bodies and 3‐methylglutaconic acid, which are normalized after recovery. A Next Generation Sequence approach with a broad gene panel designed for mitochondrial disorders revealed a novel probably pathogenic variant in homozygosity in the *LYRM4* gene [p.Tyr31Cys (c.92A>G)] with Mendelian segregation. Functional studies in the skeletal muscle confirmed a combined deficiency of the mitochondrial respiratory chain (I, II, and IV complexes). To our knowledge, this is the third case of *LYRM4* deficiency worldwide and the first with 3‐methylglutaconic aciduria, not reported in any Fe‐S cluster deficiency. Remarkably, it appears to be no neurological involvement so far, only with life‐threating acute crisis triggered by expectably benign autolimited illnesses. Respiratory chain cofactors and chaperones are a new field of knowledge and can play a remarkable effect in system homeostasis.

**Abbreviations**
3‐HMG: 3‐hydroxymethylglutaric acid
3‐MGA: 3‐methylglutaconic acid
3‐MGA‐uria: 3‐methylglutaconic aciduria
3‐OH‐IVA: 3‐hydroxyisovaleric acid
ACP: acyl carrier protein
COX: cytochrome oxidase
CS: citrate synthetase
CSF: cerebrospinal fluid
ISC: iron‐sulfur cluster
ISCU: iron‐sulfur cluster assembly scaffold protein
ISD11: ISC biogenesis desulfurase‐interacting protein 11
*LYRM4*: LYR motif‐containing protein 4
MRI: magnetic resonance imaging
NFS1: cysteine desulfurase
NGS: Next Generation Sequence
NR: normal range
OXPHOS: oxidative phosphorylation

## 1 INTRODUCTION

In the era of genomics, the classical interpretation that mitochondrial disorders comprised those with a deficiency in one or more subunits of the respiratory chain was broadened and nowadays includes defects of complex assembly, mitochondrial DNA transcription, translation and maintenance, membrane function and import, mitochondrial fusion and fission, and cofactor biosynthesis (eg, coenzyme Q_10_, lipoic acid, or iron‐sulfur clusters).^1^, ^2^ Iron‐sulfur clusters (ISCs) are crucial cofactors for mitochondrial function and facilitate the electron transport chain. More than 50 different ISCs have been identified and localized in mitochondria, nucleus, and cytosol.^3^ Mitochondrial ISCs are synthesized in the mitochondrial matrix although mitochondria also interfere in nuclear ISC assembly.^4^ Extramitochondrial functions include DNA replication, damage repair, and telomere maintenance.^5^

ISC biogenesis machinery is complex and not fully understood. It first requires the synthesis of a [2Fe‐2S] cluster by cysteine desulfurase complex (NFS1‐ISD11‐ACP), which allows cysteine to act as sulfur donor (desulfuration of cysteine to alanine) while frataxin may act as the iron donor.^3^, ^4^, ^6^, ^7^ This process relies on the action of the iron‐sulfur cluster assembly scaffold protein (ISCU) and electron chain of NAD(P)H, ferredoxin reductase, and ferredoxin.^4^, ^8^ Along with chaperones, ISCU then releases the ISC to apoproteins in an energy‐dependent reaction.^4^ Later, [4Fe‐4S] clusters will be formed, and by the action of several ISC targeting factors, they will be inserted into specific apoproteins.^4^

Twenty known genes have a direct role in mitochondrial ISC biogenesis and 17 ISC‐related genes are implicated in human disease.^9^, ^10^ For example, ISCU interacts with frataxin through ISD11, and frataxin deficiency has a negative impact on ISCs' assembly.^11^

ISD11 is a 11 kDa protein found at both mitochondria and nucleus^3^ and is codified by the well‐conserved LYR motif‐containing protein 4 gene (*LYRM4*) located in the chromosome 6p25.1. As an early intervenient in ISC biogenesis, ISD11 deficiency can have a negative interference in multiple systems and results in combined OXPHOS deficiency (MIM#615595—COXPD19). ISD11 also plays a key role in iron homeostasis as a controller of the intracellular iron trafficking, and its deficiency leads to mitochondrial iron overload.^3^, ^4^, ^11^, ^12^

To our knowledge, there are only two published patients with *LYRM4* deficiency.^9^ They were consanguineous first‐degree cousins with Lebanese/Sirian origin and a missense mutation (c.203G>T, p.R68L) leading to a nonfunctional NFS1‐ISD11 complex. The proband had a neonatal failure to thrive severe lactic acidosis, metabolic acidosis, respiratory distress, apnea, and elevated liver enzymes, all of which progressively improved, remaining healthy at the age of 20 years. His female cousin had respiratory distress right after birth and hyperlactacidemia; at 2 months of life she had a similar crisis, evolving to respiratory arrest and later died in intensive care unit. Organic acid analysis showed lactate excretion and ketosis, without other metabolites. Both had combined deficiency of complexes I, II, and III in skeletal muscle biopsy.

## 2 METHODS

Chromatography of urinary organic acids was performed by gas chromatography mass spectrometry; after acidification, salting and extraction with trimethylsilyl in a sample volume are equivalent to a creatinine volume of 5 mmol/L. Two internal standards were used (3‐phenylbutyric acid and 3‐tricarballylic acid).

Histological studies were performed on tissue from the deltoid muscle collected by open biopsy under general anesthesia. Sample was preserved in saline‐moistened gauze and immediately processed. Histological studies included modified Gomori trichrome, cytochrome oxidase (COX), and succinate dehydrogenase stains.

Spectrophotometric measurements of respiratory chain enzymes and citrate synthase were carried out in skeletal muscle homogenates as previously described,^13^ in a sample of deltoid muscle, immediately frozen at −80°C until analysis.

Genetic studies were performed by a Next Generation Sequence (NGS) approach with a gene panel designed for mitochondrial disorders including 209 nuclear genes known to be associated with mitochondrial diseases (Table S1). Genes of interest were captured using the SureSelectQXT kit (Agilent Technologies), followed by sequencing on the Illumina MiSeq platform. This study was approved by the Ethics Committee of Centro Hospitalar do Porto. Variants were filtered taking into account: (a) the type of pathogenic variant (missense, frameshift, stop‐gain or stop‐loss, and splice‐site variants), (b) in silico predictors (SIFT, PolyPhen‐2, MutationTaster) and their presence in databases (dbSNP, 1000 Genomes, HGMD professional, ClinVar, ExAC, OMIM, gnomAD), and (c) the population frequency [variants with a minor allele frequency (MAF) <1% in the 1000 Genomes Project (http://www.1000genomes.org) and Exome Variant Server databases (http://evs.gs.washington.edu) were filtered out]. Sanger sequencing was used to validate the mutation and to study Mendelian segregation in parents.

## 3 CASE REPORT

We present the case of a 4‐year‐old girl, first child of a non‐consanguineous healthy Portuguese couple, with normal growth and neurodevelopment. She had an episode of cardiorespiratory arrest at the age of 2 years after a respiratory viral infection. Microbiological studies identified respiratory syncytial virus (RSV) in respiratory secretions and Human herpesvirus 6 (HVV6) in cerebrospinal fluid (CSF). Work‐up during recovery showed a persistent increased lactate (maximum 5 mmol/L, normal range [NR] <2.0 mmol/L). Her electrocardiogram, echocardiogram, and brain magnetic resonance imaging (MRI) were normal. She was discharged with pending metabolic results and was lost in the follow‐up.

At the age of 3 years, she was admitted to our hospital after cardiac arrest following acute respiratory insufficiency. Similar to the first episode history, parents reported nasal discharge, cough, fever, and reduced appetite for the previous 4 days, with a progressive decrease of activity. That day she had been lethargic and presented with difficulty to breath prior to the cardiorespiratory arrest.

After resuscitation, apart from pulmonary auscultation with rhonchi, her physical examination was normal. Diagnostic investigation revealed persistently high lactate (2.3‐4.1 mmol/L, NR <2.0 mmol/L), normal blood and CSF counts (lactate in CSF was not performed), negative C‐reactive protein, and sterile blood culture. She had no hypoglycemia and her initially elevated liver enzymes and creatine kinase (CK) (3‐4 times the upper normal limit) rapidly returned to normal values. Chest X‐ray showed no pneumoniae and RSV was isolated in respiratory secretions. Cardiac evaluation (electrocardiogram, echocardiogram, and continuous electrocardiographic recording) was normal and an immunodeficiency was excluded (normal immunoglobulins, flow cytometry, and adequate response to tetanus and pneumococcus vaccines).

**Figure 1:**
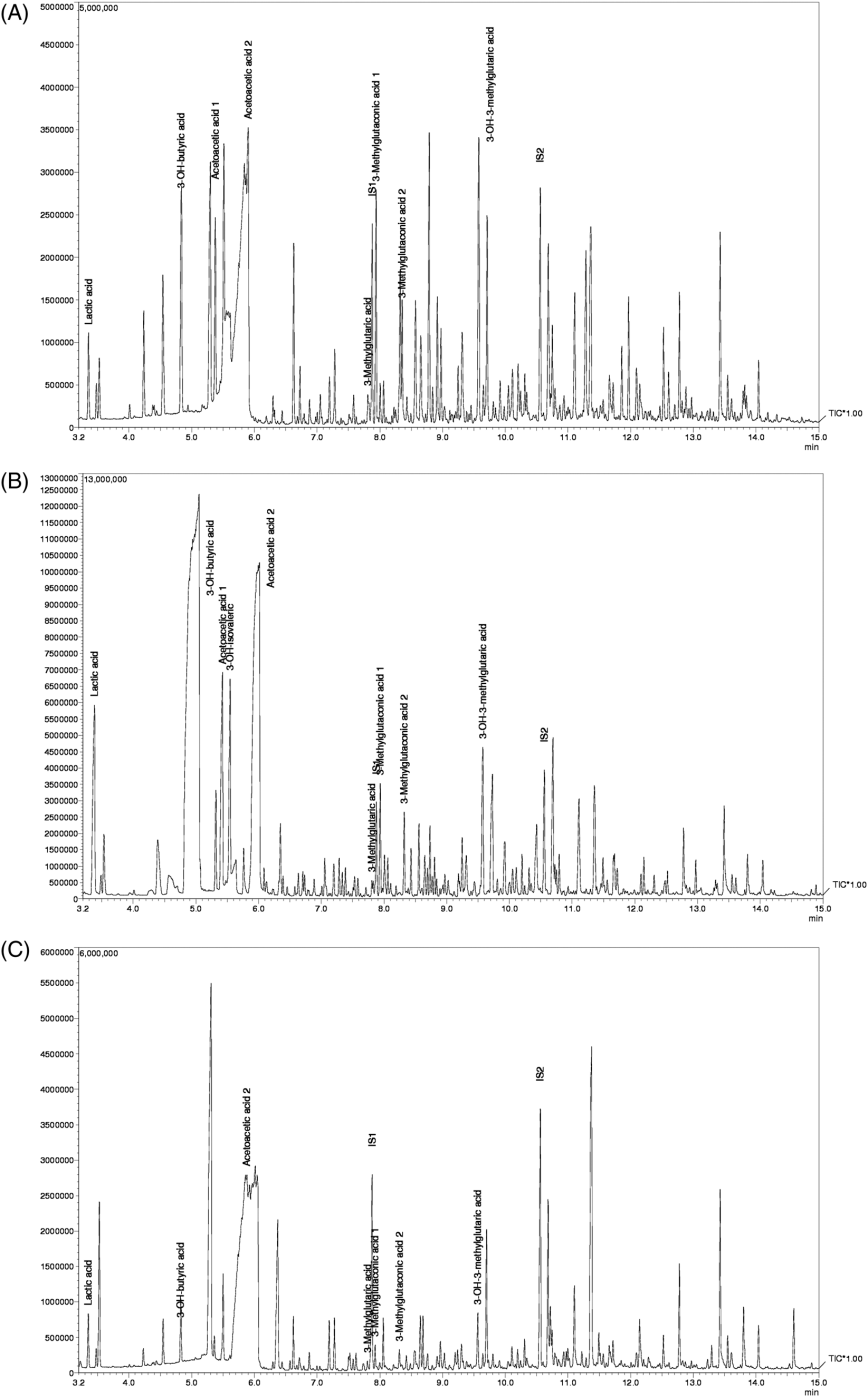
Chromatograms of urinary organic acids showing excretion of 3‐methylglutaconic acid during acute crises: A, cardiorespiratory arrest, B, during febrile mild illness without clinical deterioration, and C, during an intercrisis interval. IS1, internal standard 1 (3‐phenylbutyric acid); IS2: internal standard 2 (3‐tricarballylic acid)

Plasma amino acids were normal and acylcarnitines showed only low free carnitine (6.5 μM, NR 19‐48 μM) with normal acetylcarnitine. Urinary organic acids on admission showed ketonuria and moderate excretion of 3‐methylglutaconic acid (3‐MGA) with 154 μmol/mmol creatinine (NR <19 μmol/mmol creatinine) and mild excretion of 3‐hydroxyisovaleric acid (3‐OH‐IVA) with 85 μmol/mmol creatinine (NR 10.4‐67.0 μmol/mmol creatinine) and 3‐hydroxymethylglutaric acid (3‐HMG) with 93 μmol/mmol creatinine (NR 6.2‐49.7 μmol/mmol creatinine), as shown in Figure 1.

At this point, she enrolled in a research study for the diagnosis of mitochondrial disorders by an NGS approach. Retrieving information from the first episode showed that 2 weeks after the cardiorespiratory arrest she had mild excretion of 3‐MGA (27 μmol/mmol creatinine) accompanied by multiple other metabolites, such as 4‐hydroxyphenyllactic and 3‐hydroxyadipic acids and traces of 3‐methylglutaric, 2‐OH‐IVA, and sebacic acids.

After 5 days on mechanical ventilation and vasoactive support, she was weaned to spontaneous breathing and after 24 hours on noninvasive ventilation. Asymptomatic and with a normal neurologic examination, she was discharged after 2 weeks under carnitine per os. After recovery, lactate and organic acids completely normalized as well as carnitine levels, even after suspension of carnitine supplementation.

A close follow‐up was guaranteed. During the subsequent episode of fever with reduced oral intake, without evidence of significant general status or neurological deterioration, CK levels and liver enzymes were normal but her urinary profiling again revealed marked ketosis and mild excretion of lactate, 3‐MGA (129 μmol/mmol creatinine), 3‐OH‐IVA (162 μmol/mmol creatinine), and 3‐HMG (102 μmol/mmol creatinine) (Figure 1). She has been admitted again to the pediatric ward for two other occasions: one for fever with reduced oral intake and lethargy and another for a wheezing episode with hypoxia, both with full recovery without any complications.

During an intercritical and asymptomatic interval, metabolic re‐evaluation had no hyperlactacidemia, ketosis, elevated CK, or altered organic acid excretion (Figure 1).

At the age of 4 years, she currently attends preschool and has ballet lessons, which she completes without difficulty and both parents and patient do not report any complaints. Her physical examination remains normal.

Currently, the patient is not under any treatment. Follow‐up includes regular clinical and biochemical evaluations along with general recommendations. Special caution is given during catabolic states with admittance and intravenous fluid intake (5% dextrose in normal saline). Brain MRI evaluation was delayed pondering the general anesthetic risk vs her normal neurological status.

## 4 RESULTS

**Figure 2:**
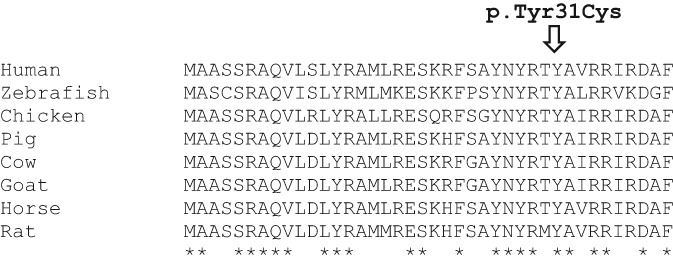
Conservation of p.Tyr31 across species. The amino acid sequence of 1‐40 of human *LYRM4* is shown and compared to the corresponding sequences of eight species. Amino acid positions identical to human genome are represented as *. The p.Tyr31Cys variant is conserved among all species examined

NGS for mitochondrial disorders revealed a probably pathogenic novel variant in homozygosity in the *LYRM4* gene [p.Tyr31Cys (c.92A>G)], with heterozygous parents for the same variant. The pathogenicity was predicted bioinformatically by SIFT, PolyPhen‐2, and MutationTaster as probably pathogenic variants. The p.Tyr31Cys variant affects an amino acid that is highly conserved in different species through evolution (Figure 2) and was absent in 100 alleles from Portuguese subjects and is not reported in public SNP databases, including dbSNP, the Exome Variant Server, and gnomAD (without any reported homozygote).

A muscle biopsy was performed. Histology showed occasional atrophy of muscle fibers and frequent accumulation of lipid droplets, without variation on fiber size or COX negative or red‐ragged fibers. Functional assay of the respiratory chain showed elevated citrate synthetase activity (354.3 nmol/min/mg, NR 85.0‐180 nmol/min/mg) and confirmed the diagnosis with the evidence of a combined deficiency of complexes I, II, and IV with 40, 23, and 31% of residual activity, respectively (Table 1).

**Table 1:** Respiratory chain functional assay in skeletal muscle

	Measured activity (nmol/min/mg NPC/CS)	Residual activity (%)	Reference values (nmol/min/mg NPC/CS)
Complex I	8.0	40	8.8‐30.8
Complex II	5.4	23	12.0‐35.0
Complex III	21.1	50	22.2‐62.2
Complex II + III	1.3	17	2.6‐12.0
Complex IV	7.1	31	11.5‐34.5

Abbreviations: CS, citrate synthetase; NPC, noncollagenous proteins.

## 5 DISCUSSION

This case broadens the phenotype knowledge on ISD11 deficiency being the third case worldwide with a novel pathogenic variant on *LYRM4*. Assumptions based on a small series must be cautious, but within these three patients, rapid clinical status deterioration with respiratory failure and arrest in the course of an infectious intercurrence appear to be a common feature. When surviving these acute crises, apparently asymptomatic periods follows. Lim et al hypothesized that hepatic cystathionase activity is prone to be lower during the neonatal period subsequent to low availability of cysteine as sulfur donor, explaining both neonatal onset and ability to survive after this period.^9^ However, this justification does not account for the late onset and repeated episodes on this patient.

As in other cases with ISD11 deficiency, hyperlactacidemia and ketosis where present, although not as elevated as reported. Combined respiratory chain deficiency is similar to findings in other patients with iron‐sulfur deficiencies.

A particular characteristic of this patient is the consistent and significant (>40 μmol/mmol creatinine) excretion of 3‐MGA during acute events, not previously reported in patients with any Fe‐S cluster deficiency. 3‐MGA‐uria can be found in various inborn metabolic disorders commonly along with excretion of other metabolites (fatty acid oxidation disorder, methylmalonic and propionic aciduria, glycogen storage disease, or urea cycle disorder). It can be a feature of mitochondrial disorders present in about 10% of patients,^14^ but usually is not the prominent hallmark of disease and excretion is only slightly elevated and as an accompanying finding.^15^ It represents not an impairment on the leucine pathway but an alternative route for acetyl‐CoA degradation.^16^

Inborn errors of metabolism with 3‐MGA‐uria as discriminative feature (isolated, significantly or repeatedly elevated) can be classified as primary or secondary 3‐MGA‐uria.^15^ Primary 3‐MGA‐uria is caused by 3‐methylglutaconyl‐CoA hydratase deficiency, an inborn error of the leucine pathway. Known diseases with secondary 3‐MGA‐uria can be due to the following^14^, ^15^, ^17^, ^18^, ^19^: (a) defective phospholipid remodeling: Barth syndrome (*TAZ* gene) and MEGDEL syndrome (*SERAC1* gene); (b) mitochondrial membrane‐associated disorder: Costeff syndrome (*OPA3*), TMEM70 defect (*TMEM70*), and DCMA syndrome (*DNAJC19*); (c) unknown mechanism: Sengers syndrome (*AGK*), CLPB deficiency (*CLPB*), TIMM50 deficiency (*TIMM50*), HTRA2 defect (*HTRA2*), and short‐chain enoyl‐CoA hydratase deficiency (*ECHS1*).

This is the first case of Fe‐S cluster with 3‐MGA‐uria, extending the differential diagnosis of 3‐MGA‐uria as the presenting feature.

Being a disorder related to iron metabolism, with the possibility to induce iron overload on tissues and storage depletion, evaluation of ferritin, transferrin, soluble transferrin receptor, or hepcidin may help understand the impact on the dynamics of iron metabolism, despite no evidence of impaired iron homeostasis in our patient so far.

Since cysteine acts the major sulfur donor, supplementation with this nonessential amino acid can be beneficial, especially during periods of higher energy requirement. The rationale for treatment with l‐cysteine or *N*‐acetylcysteine is supported by studies on patients with mitochondrial disorders with some benefit in those with translation‐related defects.^20^ It can be expected that a corresponding reduction in iron overload diminishes its deleterious effects.

Raised awareness and a low threshold to hospital admittance in patients with ISD11 deficiency may be crucial to improve survival. Routine neurological and neurodevelopment evaluation must be granted.

This case illustrates the importance of metabolic work‐up to be performed as soon as possible, while the patient is acutely ill, as it may be a unique opportunity to diagnose a critical condition. Moreover, life‐threatening events disproportional to symptoms and medical history should elicit the possibility of energy‐related inherited metabolic disorder. Acute presentations of mitochondrial disorders will become more common with the improved diagnostic yield of genetic studies.

## AUTHOR CONTRIBUTIONS

M.P.C. was involved in patient diagnosis, literature review, and drafting of the manuscript. J.C. was involved in patient follow‐up and investigation, and drafting of the manuscript. A.D. worked on chromatography of urinary organic acids, interpretation, and figure construction. C.N. worked on genetic studies and interpretation. A.B. and E.M. were involved in patient's work‐up and did critical manuscript review. L.V. worked on functional and genetic studies interpretation and did critical manuscript review.

## CONFLICT OF INTEREST

The authors declare that they have no conflict of interest.

## COMPLIANCE WITH ETHICAL STANDARDS

The study “Genetic Defects of Mitochondrial Diseases: a Next Generation Sequencing Approach” (FCT PTDC/DTP‐PIC/2020/2014) was approved by the Ethics Committee of Centro Hospitalar do Porto (REF. 2016.194(164‐DEFI/153‐CES). Informed consent from patient's parents was obtained.

## Supporting information


**Table S1** Nuclear genes involved in mitochondrial disorders
