# Supplementary material for: Iron‐sulfur cluster ISD11 deficiency (LYRM4 gene) presenting as cardiorespiratory arrest and 3‐methylglutaconic aciduria
Source: JIMD Rep. 2019 Jul 24;49(1):11–6. doi: 10.1002/jmd2.12058 (PMC6718106; doi:10.1002/jmd2.12058)
Supplement: Supplementary file 1 — Table S1 Nuclear genes involved in mitochondrial disorders [file JMD2-49-11-s001.docx]

Table 2. Nuclear genes involved in mitochondrial disorders

| OXPHOS subunits | |
| --- | --- |
| Complex I | *NDUFS1; NDUFS2; NDUFS3; NDUFS4; NDUFS6; NDUFS7; NDUFS8; NDUFV1; NDUFV2; NDUFA1; NDUFA10; NDUFA11; NDUFA12; NDUFA2; NDUFA9; NDUFB3; NDUFB9* |
| Complex II | *SDHA; SDHB; SDHC; SDHD* |
| Complex III | *CYC1; LYRM7; UQCRQ; UQCRC2; UQCRB* |
| Complex IV | *COX5A; COX5B; COX4I2; COX6A1; COX6B1; COX7B; NDUFA4* |
| Complex V | *ATP5A1; ATP5E* |
| OXPHOS assembly factors | |
| Complex I | *ACAD9; FOXRED1; NDUFAF1; NDUFAF2; NDUFAF3; NDUFAF4; NDUFAF5; NDUFAF6; NUBPL* |
| Complex II | *SDHAF1; SDHAF2* |
| Complex III | *BCS1L; HCCS; TTC19* |
| Complex IV | *COA3; COA5; COX10; COX15; COX14; COX20; FASTKD2; SCO1; SCO2; SURF1* |
| Complex V | *ATPAF2; TMEM70* |
| mtDNA maintenance | *C10ORF2; DNA2; DGUOK; FBXL4; POLG; MGME1; MPV17; POLG2; SLC25A4; SUCLA2; SUCLG1; TYMP; TK2; RRM2B* |
| Mitochondrial translation | *AARS2; C12ORF65; CARS2; DARS2; EARS2; ELAC2; FARS2; GFM1; GTPBP3; HARS2; IARS2; KARS; LARS; LARS2; LRPPRC; MARS2; MRPL23; MRPL3; MRPL44; MRPL50; MRPL57; MRPS16; MRPS22; MTFMT; MTO1; MTPAP; NARS2; PARS2; PNPT1; PUS1; RARS2; RMND1; RNASEH1; SARS2; TACO1; TARS2; TRMU; TRNT1; TSFM; TUFM; VARS2; WARS2; YARS2* |
| Membrane function and import | *AGK; C19ORF12; CISD2; DNAJC19; DNM1L; GDAP1; GFER; MFF; MFN1; MFN2; MICU1; MPC1; OPA1; SERAC1; SLC25A22; SLC25A3; SLC25A42; SLC4A3; SLC52A1; SLC52A2; SLC52A3; TAZ; TIMM8A; TMEM126A; TMEM126B* |
| Cofactor biosynthesis (CoQ10, lipoic acid, FeS clusters, others) | *ABCB7; ADCK3; BOLA3; COQ2; COQ4; COQ6; COQ9; FDX1L; GLRX5; IBA57;ISCU; LIAS; LIPT1; LYRM4; NFU1; PDSS1; PDSS2; TPK1* |
| Others | *ACO2; AIFM1; ALAS2; ALG13; APTX; AUH; C9ORF116; CHKB; CLN3; CLN5; CLN6; CLN8; CLPB; CTSD; CWF19L1; DLAT; DLD; DNAJC5; DOLK; ECHS1; ETFA; ETFB; ETFDH; ETHE1; FLAD1; HACE1; HIBCH; KCTD7; KIF5A; MEGF10; MFSD8; MPI; NOTCH3; OPA3; PC; PCK2; PDHA1; PDHB; PDHX; PDP1; PLD1; PMM2; PPT1; SLC19A3; SPATA5; TPP1; WDR45; WFS1* |
